# Association of Serum Magnesium with Blood Pressure in Patients with Hypertensive Crises: A Retrospective Cross-Sectional Study

**DOI:** 10.3390/nu13124213

**Published:** 2021-11-24

**Authors:** IfeanyiChukwu O. Onor, Lashira M. Hill, Modupe M. Famodimu, Mallory R. Coleman, Carolkim H. Huynh, Robbie A. Beyl, Casey J. Payne, Emily K. Johnston, John I. Okogbaa, Christopher J. Gillard, Daniel F. Sarpong, Amne Borghol, Samuel C. Okpechi, Ifeyinwa Norbert, Shane E. Sanne, Shane G. Guillory

**Affiliations:** 1 CardioRenal Research Group (CRRG), College of Pharmacy, Xavier University of Louisiana, 1 Drexel Drive, New Orleans, LA 70125, USA; lashirahill@gmail.com (L.M.H.); modupealabi@gmail.com (M.M.F.); malloryroy89@gmail.com (M.R.C.); carolkimh@gmail.com (C.H.H.); caseyjpayne@msn.com (C.J.P.); ejohnston831@gmail.com (E.K.J.); jokogbaa@xula.edu (J.I.O.); cgillard@xula.edu (C.J.G.); dsarpong@xula.edu (D.F.S.); aborghol@xula.edu (A.B.); ifeyinwanorbert@gmail.com (I.N.); 2 Department of Medicine, Louisiana State University Health Sciences Center School of Medicine, 1542 Tulane Avenue, New Orleans, LA 70112, USA; ssann1@lsuhsc.edu (S.E.S.); sguil1@lsuhsc.edu (S.G.G.); 3 Department of Pharmacy, University Medical Center New Orleans, 2000 Canal Street, New Orleans, LA 70112, USA; 4 Pennington Biomedical Research Center, 6400 Perkins Road, Baton Rouge, LA 70808, USA; Robbie.Beyl@pbrc.edu; 5 Center for Minority Health and Health Disparities Research and Education, College of Pharmacy, Xavier University of Louisiana, 1 Drexel Drive, New Orleans, LA 70125, USA; 6 Department of Biochemistry and Molecular Biology, Louisiana State University School of Medicine and Health Sciences Center, 1901 Perdido Street, New Orleans, LA 70112, USA; sokpec@lsuhsc.edu

**Keywords:** magnesium, blood pressure, hypertensive crises

## Abstract

The role of magnesium in blood pressure has been studied among hypertensive patients; however, there is a dearth of studies exploring the role of magnesium in hypertensive crises. The primary objective of this study was to evaluate the relationship between serum magnesium and blood pressure in patients with hypertensive crises. This was a single-center, retrospective, chart review, cross-sectional study of patients with hypertensive crises. Patients were included if they were eighteen years of age or older, with an international classification disease ninth revision (ICD-9) code of 401.9 (hypertensive crises: emergency or urgency) and a documented magnesium level on their electronic medical record. The primary outcome of the study was the correlation between serum magnesium and blood pressure (systolic blood pressure and diastolic blood pressure) in patients with hypertensive crises. Two hundred and ninety-three patients were included in the study. The primary outcome result showed that serum magnesium was positively correlated with systolic blood pressure (r = 0.143, *p* = 0.014), but not diastolic blood pressure. Conclusion: This study found a significant positive association between magnesium and systolic blood pressure, but not diastolic blood pressure, among patients with hypertensive crises. This positive association of serum magnesium with systolic blood pressure was maintained after adjusting for covariates. This study’s findings suggest a potential role of magnesium in blood pressure among patients with hypertensive crises.

## 1. Introduction

Hypertension is a condition characterized by elevation in the systolic blood pressure (SBP) and/or diastolic blood pressure (DBP) [1,2]. Clinical practice guidelines define hypertension using different cutpoints, as either SBP greater than or equal to 130 mmHg and/or DBP greater than or equal to 80 mmHg [3], or SBP greater than or equal to 140 mmHg and/or DBP greater than or equal to 90 mmHg [4,5]. The global prevalence of hypertension in adults is between 30 and 45% [5]. The prevalence of hypertension among US adults varies according to the clinical practice guideline cutpoints used to categorize blood pressure, with an overall prevalence of hypertension among US adults between 32 and 46% [3,4]. Hypertension remains a major risk factor for cardiovascular diseases (hemorrhagic stroke, ischemic stroke, myocardial infarction, angina, heart failure, peripheral artery disease, and aortic aneurysm), end-stage renal disease, death, and disability [1,2,3,4,5,6]. Beyond cardiovascular disease, hypertension has also been associated with declines in both cognitive function and psychological health [7,8,9]. Hypertensive crises is defined as SBP greater than 180 mmHg and/or DBP greater than 120 mmHg [3,4]. Hypertensive crises can be further classified into hypertensive urgency (absent evidence of target organ damage) and hypertensive emergency (evidence of target organ damage exists) [3,4]. Although hypertensive urgency reflects a marked elevation in blood pressure, it can be managed by increasing or optimizing the dose of oral antihypertensive agents. Hypertensive emergency, however, is characterized via target organ damage and is associated with a 1-year mortality rate of >79%, thus necessitating swift blood pressure reduction with intravenous antihypertensive agents to prevent sustained deterioration of target organ damage [3,4].

Magnesium is the second most abundant intracellular cation after potassium and the fourth most abundant cation in the body [10,11,12]. In adult humans, total body magnesium store is approximately 24 g with 99% intracellular distribution (bone (53%), muscle (27%), and soft tissue (19%)) and 1% in the extracellular space (serum and erythrocytes) [10,11]. The normal total serum concentration is in the range of 1.7–2.6 mg/dL (0.7–1.1 mmol/L) [10]. The normal serum magnesium range represents approximately 0.3% of total body magnesium and may not precisely reflect the total magnesium status [11,13]. Serum magnesium homeostasis is regulated by the interplay between gastrointestinal transport, renal exchange, and bone exchange [10]. Magnesium is involved in a plethora of physiologic processes, namely, intracellular signaling, serving as a cofactor for DNA and protein synthesis, oxidative phosphorylation, cardiac excitability, vasomotor tone, blood pressure homeostasis, neuromuscular conduction, and bone formation [10,11,12].

Multiple clinical trials/reviews have shown, albeit inconsistently, that magnesium deficiency (serum and/or tissue) exist to some extent in hypertensive subjects, with decreased magnesium levels linked to undesirable effects on blood pressure [14,15,16,17,18,19]. Although magnesium has been posited to modulate blood pressure regulation, the precise mechanism of altered magnesium metabolism in hypertensive individuals has not been effectively elucidated in the literature [14]. The most popular proposed mechanism of the effect of magnesium on blood pressure is that magnesium acts as a natural calcium antagonist on most types of calcium channels in vascular smooth muscles, thus reducing arterial blood pressure through a lowering of peripheral and cerebral vascular resistance [14]. More specifically, magnesium acting as a calcium antagonist produces endothelial-dependent vasodilation, and reduces blood pressure via increases in extracellular magnesium and reductions in calcium influx [13,14,20,21]. Magnesium has also been shown to produce vasodilation by increasing prostaglandin E—a vasodilator and platelet inhibitor [20,21]. Magnesium is also an essential cofactor for delta-6-desaturase enzyme, which generates gamma linolenic acid (a precursor to prostaglandin E) from linoleic acid [20,21]. Additionally, a robust interaction has been found between magnesium and other electrolytes (potassium, calcium, and sodium) in blood pressure reduction, with low intracellular sodium and calcium and high intracellular magnesium and potassium shown to improve blood pressure [20,21].

Multiple observational clinical studies and a meta-analysis have evaluated the relationship between serum magnesium and blood pressure in patients with and without hypertension [15,17,18,19,22,23,24,25,26,27,28]; however, there is a dearth of studies that have evaluated the association of serum magnesium and blood pressure in patients with hypertensive crises. The available publications performed tests of association (correlation, risk ratios, odds ratio, and hazard ratios) between magnesium and either blood pressure or hypertension [15,17,18,19,22,23,24,25,26,27,28]. The ten studies that performed a test of association between magnesium and either blood pressure or hypertension have reported conflicting evidence: six studies found a significant negative association [17,18,19,24,25,28], three studies found no significant relationship [15,23,27], and one study found a significant positive correlation between serum magnesium and blood pressure in women only [22]. A meta-analysis found no significant association between serum magnesium and blood pressure, although there was a trend towards negative association between serum magnesium and blood pressure (risk ratio (RR) = 0.91, 95% CI: 0.80–1.02) [26]. Similarly, the impact of magnesium supplementation on blood pressure has been studied extensively. Nine of the ten studies (clinical trials, meta-analyses, and Cochrane Review) reviewed mostly reported a positive role of magnesium supplementation in lowering SBP, DBP, or both [26,29,30,31,32,33,34,35,36], and only one study found no significant effect of magnesium supplementation on blood pressure [37]. This recurring positive impact of magnesium supplementation in lowering blood pressure served as the foundational rationale for our study evaluating whether serum magnesium is a factor that contributes to the dysregulated high blood pressure seen in patients with hypertensive crises. We hypothesized that low serum magnesium will be significantly associated with blood pressure (SBP and DBP) in patients with hypertensive crises.

The primary objective of this study was to evaluate the relationship between serum magnesium and blood pressure (SBP and DBP) in hypertensive crises. The secondary objectives were to evaluate the association between serum calcium, corrected calcium, and serum potassium on blood pressure in patients with hypertensive crises, and to determine the influence of covariates on the relationship between serum magnesium and blood pressure in patients with hypertensive crises. Given that hypertensive crisis is a state of dysregulated high blood pressure, it is vital to study the role that factors such as serum magnesium play in the etiology of hypertensive crises. Data from these studies can be hypothesis-generating and provide evidence for studying potential innovative therapies to manage hypertensive crises, such as serum magnesium-modifying therapies.

## 2. Materials and Methods

This was a single-center, retrospective, chart review, cross-sectional study conducted at the Interim Louisiana State University Hospital (ILH)—New Orleans, Louisiana. The study data were derived from patients who were admitted to ILH with hypertensive crises during a 2-year period from July 2012 to July 2014. This study was approved by the ILH Research Review Committee (RRC) and Xavier University of Louisiana Institutional Review Board (IRB).

Patients eighteen years of age or older with an international classification disease ninth revision (ICD-9) code of 401.9 (hypertensive crises: emergency or urgency) and a documented serum magnesium level on their electronic medical record (during the hypertensive crises hospital admission) were included in the study. Hypertensive crisis was defined as systolic blood pressure (SBP) greater than 180 mmHg and/or diastolic blood pressure (DBP) greater than 120 mmHg [3,4]. Patients identified as having hypertensive crises based on ICD-9 codes were confirmed to have either SBP greater than 180 mmHg and/or DBP greater than 120 mmHg. Hypertensive crisis was classified as either hypertensive urgency (absence of acute or on-going target organ damage) or hypertensive emergency (presence of acute or on-going target organ damage). Target organ damage by system included neurologic (hypertensive encephalopathy, intracranial hemorrhage, acute ischemic stroke), cardiac (acute myocardial infarction, acute left ventricular failure, unstable angina, dissecting aortic aneurysm), and renal (acute kidney injury). All diagnoses of target organ damage were confirmed with both clinical findings (laboratory results, imaging, signs, and symptoms) from the patients and the physician’s diagnosis, documented on the patients’ problem list. Hypertensive encephalopathy diagnosis was verified based on physical exam findings of headache and altered levels of consciousness. Diagnosis of intracranial hemorrhage and acute ischemic stroke was confirmed using a computed tomography (CT) scan or magnetic resonance imaging of the head with or without contrast, and was performed on patients with neurologic symptoms, which included changes in mental status or focal neurologic signs indicative of cerebrovascular accident or hemorrhage. Unstable angina diagnosis was made clinically and confirmed with documented new or sudden chest pain, while myocardial infarction diagnosis was confirmed with elevated serum troponin levels and electrocardiogram (EKG) findings. Acute left ventricular failure was diagnosed with echocardiographic findings of a decreased ejection fraction less than 40%, as well as physical exam findings of elevated jugular venous pressure (distension), crackles, or edema. Diagnosis of dissecting aortic aneurysm was confirmed via imaging studies revealing wide mediastinum on chest X-ray and/or chest CT scan with or without contrast. Acute kidney injury was defined as a serum creatinine (SCr) greater than 2 mg/dL, which is new-onset in the absence of prior renal disease and/or increases in SCr of 0.5 mg/dL or greater.

Patients were excluded if they had conditions interfering with serum magnesium levels, such as chronic kidney disease (CKD) stages 4 and 5, end-stage renal disease (ESRD), hepatic cirrhosis, pheochromocytoma, chronic diarrhea, and hyperaldosteronism. Additionally, patients who received vasopressors or inotropes (including milrinone, dobutamine, epinephrine, norepinephrine, dopamine, phenylephrine, or vasopressin) during the hospital encounter were excluded from the study.

All patient data were obtained from ILH’s electronic medical record. The following demographic data were collected: age, sex, race, body mass index (BMI), and history of diabetes mellitus. The key variables obtained for our primary and secondary outcome analyses include serum magnesium (mg/dL), serum calcium (mg/dL), serum potassium (mEq/L), SBP (mmHg), and DBP (mmHg). These variables were obtained at a time closest to that at which blood pressure was measured in the first documented hypertensive crises during the hospital encounter, denoted as “at crises”. The Synchron^®^ systems detection method and the Beckman DXC 600 instrument were used to measure the serum magnesium, serum calcium, and serum potassium levels. Additionally, maximum and minimum values of SBP and DBP were recorded within 24 h of the first recorded hypertensive crises’ blood pressure. All SBP and DBP measurements were taken at intervals specified by the medical unit/floor whereat each patient was admitted. Corrected calcium (mg/dL) was calculated using the formula: corrected calcium = patient’s measured serum calcium in mg/dL + (0.8 × (4 gm/dL—patient’s measured albumin in gm/dL)). The corrected calcium was only calculated for patients whose serum albumin was less than 4 gm/dL. Additional independent variables collected included home and hospital use of blood pressure medications (inclusive of all blood pressure medication classes (for example, calcium channel blockers) grouped in the electronic health record), at home use of proton pump inhibitors, home use of oral magnesium, hospital use of intravenous magnesium, and albumin levels (gm/dL).

The primary outcome of the study was the correlation between serum magnesium and blood pressure (SBP and DBP) in patients with hypertensive crises. The secondary outcomes were the associations of serum calcium, corrected calcium, and serum potassium with blood pressure in patients with hypertensive crises, and the relationship between serum magnesium and blood pressure when adjusting for covariates (age, sex, race, BMI, history of diabetes mellitus, use of proton pump inhibitors at home, use of blood pressure medications at home or hospital, use of oral magnesium at home, use of intravenous magnesium at hospital, serum calcium at crises, corrected calcium at crises, and serum potassium at crises). We selected these covariates to control for their potential confounding effects on the results of our study, since the covariates were variables that could potentially affect SBP, DBP, and serum electrolyte levels. An additional exploratory outcome was the correlation of serum magnesium, serum calcium, corrected calcium, and serum potassium with the two independent variables SBP and DBP, measured at different time points.

We performed a power analysis (based on findings from prior studies) on our key variables for the primary outcome of the study (serum magnesium, SBP, and DBP) [19,29,31]. Based on these studies, we estimated that the R^2^ (coefficient of determination) for the linear regression between serum magnesium and either SBP or DBP would range between of 0.06 and 0.56. Our power analysis revealed that the target sample size for this study will range between 140 and 180 subjects, to give us a power of 0.80 at a significance level of 5%.

Descriptive statistical analysis was performed on demographic characteristics. Measures of central tendency were obtained for continuous measures and frequency distribution for categorical measures. Simple linear regression was conducted to assess the correlation (r) and coefficient of determination (R^2^) between serum magnesium, serum calcium, corrected calcium, and serum potassium in relation to blood pressure (SBP and DBP). A linear model analysis was conducted to evaluate the association between serum magnesium (and other electrolytes) and blood pressure (SBP and DBP) at the time of hypertensive crises while adjusting for covariates. Statistical analyses were performed using SAS^®^ version 9.4. A *p*-value of less than 0.05 was considered statistically significant in the two-tailed test.

## 3. Results

**Figure 1:**
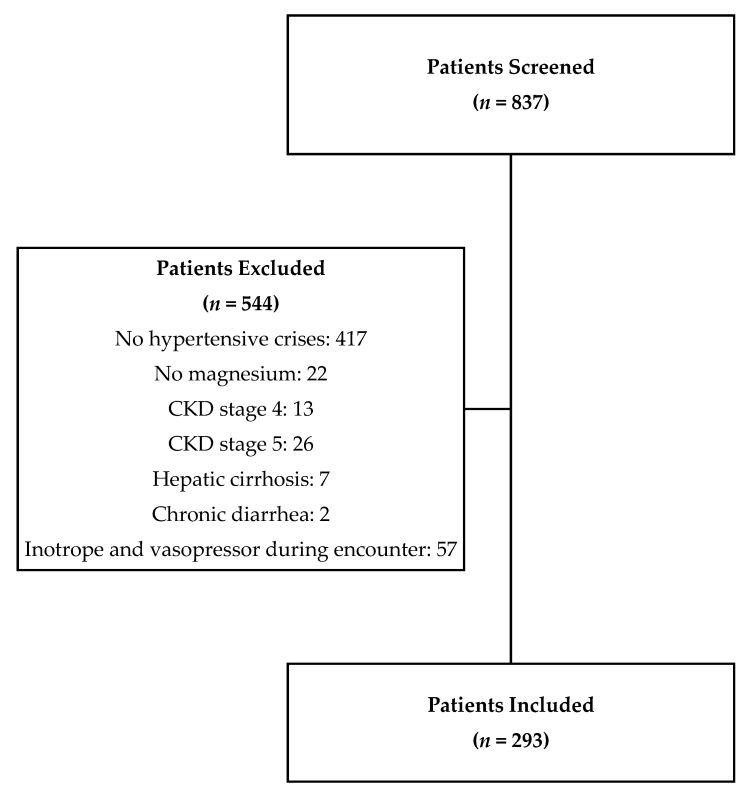
Flow chart of inclusion in study cohort. CKD—chronic kidney disease.

Eight-hundred and thirty-seven patients who had a serum magnesium level and an ICD-9 code of 401.9, or a diagnosis of hypertensive crises, hypertensive urgency or hypertensive emergency, were identified via ILH’s electronic medical records (Figure 1). In total, 544 patients were excluded after reviewing and applying the study inclusion/exclusion criteria, and 293 patients were included in the statistical analysis (Figure 1). Baseline demographics are presented in Table 1. The majority of patients were African Americans (72.7%) and the mean age of patients in the study was 56.7 years. Hypertensive urgency (75.1%) was greater in prevalence than hypertensive emergency (24.9%) among all hypertensive crisis diagnoses. Nearly 35% of patients had diabetes mellitus and the average BMI of the study population was 30.6 kg/m^2^.

The primary outcome result is presented in Table 2, and this reveals that serum magnesium was positively correlated (r = 0.143, *p*-value = 0.014) with SBP at crises, but not DBP at crises. The coefficient of determination (R^2^) reveals that 2% of the variability in SBP at crises can be attributed to serum magnesium. Besides serum magnesium, serum calcium was the only additional electrolyte significantly correlated with SBP at crises. Serum calcium was positively correlated with SBP at crises, with 3.5% of the variability in SBP at crises attributable to serum calcium. When evaluating DBP at crises, no electrolyte was significantly correlated with DBP at crises; however, serum potassium showed a slight trend towards a negative correlation with DBP at crises.

Table 3 assessed the relationship with serum magnesium and both SBP and DBP at crises when adjusting for covariates. The results show that as serum magnesium increased by 1 mg/dL, SBP at crises increased 11.25 mmHg after adjusting for covariates in the model (*p*-value = 0.017). Although a significant association was found with serum magnesium and SBP at crises after adjusting for covariates, no significant association was found with serum magnesium and DBP at crises after adjusting for covariates in the model.

Variables that were significantly associated with SBP at the time of hypertensive crises was assessed using a linear model (adjusted for covariates), and the results are displayed on Table 4. When adjusting for covariates, serum magnesium, serum calcium, corrected calcium, and the use of proton pump inhibitors independently emerged as the best variables that were significantly associated with SBP at crises, with all showing a positive relationship with SBP at crises except for corrected calcium level, which showed a negative relationship with SBP at crises. Similarly, Table 5 displays the variables that were significantly associated with DBP at time of hypertensive crises using a linear model (adjusted for covariates). In the model, after adjusting for covariates, serum calcium, corrected calcium, and age independently emerged as the best variables that were significantly associated with DBP at crises. Serum calcium had a positive relationship with DBP at crises in the model, while corrected calcium and age had a negative association with DBP at crises in the model. All covariates were held constant during the linear model analysis, except the independent and dependent variables being compared at each point.

Table 6, Table 7, Table 8 and Table 9 display the results of the correlation matrix analyses of serum magnesium, serum calcium, corrected calcium, and serum potassium with the two independent variables SBP and DBP measured at different time points. Table 6 shows that serum magnesium was significantly correlated only with SBP at time of crises. The results displayed in Table 7 show that serum calcium was significantly correlated with SBP and DBP measured at different time points, with the exception of DBP measured at the time of crises, which did not show significant correlation with serum calcium. The corrected calcium results displayed in Table 8 reveal that corrected calcium was only significantly correlated with minimum SBP and minimum DBP measured within 24 h of the first hypertensive crises diagnosis. Lastly, serum potassium was only significantly correlated (negative correlation) with minimum SBP and minimum DBP measured within 24 h of the first hypertensive crises diagnosis.

## 4. Discussion

This study contributes much new knowledge on the role of magnesium in patients with hypertensive crises—a population where the role of magnesium has been sparsely evaluated. The primary objective of our study was to evaluate the relationship between magnesium and blood pressure (SBP and DBP) in patients with hypertensive crises.

Using correlation (Table 2) and linear regression analyses (Table 3), our study found a significant positive correlation between serum magnesium and SBP at crises, but no significant relationship was found between serum magnesium and DBP at crises. This finding of positive correlation between serum magnesium and SBP in our study conflicts with the majority of studies that assessed serum magnesium and SBP, which have predominantly shown a significant negative correlation [15,17,18,19,22,25,28]. Among the seven studies that evaluated the linear relationship (correlation coefficient) between magnesium and either SBP or DBP [15,17,18,19,22,25,28], the majority (*N* = 5 out of 7, 71.4%) showed a negative relationship between serum magnesium and SBP [17,18,19,25,28]. One of the studies showed no significant relationship between serum magnesium and SBP [15], while one study by Rinner et al. approximated our study finding, and revealed a positive relationship between serum magnesium and SBP in women, but not in men [22]. With respect to the relationship between serum magnesium and DBP, the majority of the studies (*N* = 4 out of 7, 57%) have shown a non-significant relationship, which is consistent with our study findings [15,17,22,28]. However, three studies found a negative correlation between serum magnesium and DBP, which contradicts our study results [18,19,25]. Our study finding is therefore consistent with most of the available literature showing no strong relationship between serum magnesium and DBP; however, our study finding conflicts with the predominant negative linear relationship observed between serum magnesium and SBP in studies. Altogether, our study’s detection of a significant positive association between serum magnesium and SBP in patients with hypertensive crises indicates that serum magnesium may play an important role in the dysregulated blood pressure observed in patients with hypertensive crises. Our study finding of increasing SBP with increasing magnesium conflicts with the prevailing postulated mechanism that magnesium reduces arterial blood pressure [14]. It is reasonable to consider that our study may have derived disparate results (especially between magnesium and SBP) from the literature due to potential errors arising from selection bias, confounding variables, the heterogeneity of the patient populations included in the studies, and the majority of the studies reviewed from the literature being non-experimental association studies [17,18,19,25,28].

When assessing the relationship between the additional electrolytes reviewed (calcium, corrected calcium, and potassium) and blood pressure, we observed that serum calcium was significantly positive correlated with SBP at crises, but not DBP at crises. After adjusting for covariates (Table 4 and Table 5), we found a positive relationship between serum calcium and both SBP and DBP at crises. Similar to our study finding, the positive association of serum calcium with both SBP and DBP has been reported in several studies that evaluated the linear relationship between serum calcium and blood pressure [19,38,39,40,41,42]. It is important to reinforce that our study found a relationship between serum calcium and both SBP and DBP at crises after adjusting for covariates; however, the correlation analysis without adjustment for covariates showed a significant positive correlation between serum calcium for SBP at crises, but not DBP at crises. The suggested mechanism of the role of calcium in blood pressure regulation is through the mediation of the vasoconstriction of vascular smooth muscle, alterations in extracellular binding of calcium, the interaction between serum calcium and other cations such as sodium, potassium and magnesium, renal vasoconstriction causing kidney dysfunction, and hyperactivity of the renin–angiotensin system caused by hyperparathyroidism [43,44]. Corrected calcium was not significantly correlated with either SBP or DBP at crises in the simple linear regression result (Table 2); however, after adjusting for covariates (Table 4 and Table 5), we found a significant negative relationship between corrected calcium and both SBP and DBP at crises. This indicates that there was a statistically significant divergent relationship in the linear model analysis between serum calcium and SBP/DBP at crises (positive association), and corrected calcium and SBP/DBP at crises (negative association). There is no biologically plausible explanation for this divergent association; however, the adjustment for covariates and the numerical difference in the number of patients with corrected calcium (*N* = 207) versus serum calcium (*N* = 293) may have led to the divergent direction of the relationship. Two hundred and seven patients had corrected calcium values because corrected calcium was only calculated for patients who had serum albumin levels less than 4 gm/dL. In the exploratory results, we found that corrected calcium was positively associated with both minimum SBP and DBP within 24 h of hypertensive crises. All the data with corrected calcium should be interpreted cautiously, given that we did not include 86 missing data points from among patients who did not have serum albumin levels less than 4 gm/dL. Potassium was also not significantly correlated with either SBP or DBP at crises, although there was a trend towards a negative correlation between potassium and DBP at crises. This finding is inconsistent with prior studies, which have predominantly shown a negative correlation between serum potassium and both SBP and DBP [22,45,46,47]. In the exploratory results (Table 6, Table 7, Table 8 and Table 9), we found that serum potassium was negatively associated with both minimum SBP and DBP within 24 h of hypertensive crises. Calcium and potassium were measured because these electrolytes have been previously associated with blood pressure, especially in concert with magnesium [3,16,18,19,20,21,22]. We also found an incidental significant positive association between the use of home proton pump inhibitors and SBP at crises—indicating that use of home proton pump inhibitors is associated with increasing SBP. We found no literature supporting an association (negative or positive) between proton pump inhibitors and hypertension; however, there are studies documenting an association between proton pump inhibitor use and hypomagnesemia [10,48]. It is, however, unclear whether the association between the use of home proton pump inhibitors and increased SBP is derived from selection bias (more hypertensive crises patients being prescribed proton pump inhibitors) or from the indirect effects of proton pump inhibitors in lowering serum magnesium. Given that we did not collect sufficient variables for statistical and sensitivity analyses, our study is limited in its ability to explain this incidental finding of association between the use of home proton pump inhibitors and increased SBP at crises.

The strengths of our study include our use of statistical tests that explored associations between variables, the pilot nature of our study, and the attainment of our study’s desired sample size. We performed regression analysis to examine the association of magnesium and other electrolytes with blood pressure in hypertensive crises. The findings from the regression analysis provide us with useful information on the relationship between electrolytes and variables. This study is also a pilot/exploratory study, and is thus a hypothesis-generating study and can provide population estimates to help determine the appropriate sample size to study the effect of magnesium on hypertensive crises in future studies. Lastly, our study reached and exceeded our desired sample size, which decreased the probability of type II errors and improved the probability of detecting significant differences that may exist in the true population of patients from which our sample population was obtained.

Several limitations impacting internal and external validity apply to our study. First, our study was a single-center study, and this limits the generalizability of our study to patients across institutions. Thus, the findings from this single-center study should be extrapolated cautiously to individual patients and patient populations with hypertensive crises. This study was a retrospective study, so variables available from the electronic medical record were not collected uniformly at specific times, as would be the case in a prospective study. Given the retrospective and non-randomized design of our study, notable variables were not collected or accounted for, which may confound our findings. Some of the potential confounding variables not accounted for include, but are not limited to, the use of calcium supplements, use of vitamin D, use of magnesium-containing antacids, use of diuretics, and renal function markers (SCr or estimated glomerular filtration rate). Our study was also a non-interventional/non-experimental study, which impacts the internal validity and limits our study’s ability to assess causation. Our study was not a randomized study, and confounding variables may have impacted our results. Sodium’s relationship with blood pressure was not assessed in our study, which is a limitation, given that an interaction has been found between magnesium and other electrolytes (potassium, calcium, and sodium) in blood pressure reduction [20,21]. Lastly, our study may be susceptible to selection bias as a source of error, due to the study inclusion/exclusion criteria and the unique demographic distribution of the study patients at our hospital compared to our study’s broad target population of patients with hypertensive crises. Another important study limitation is that magnesium is predominantly an intracellular cation, thus, the serum magnesium obtained from the electronic hospital records may not be a good reflection of patients’ magnesium stores [10,11].

## 5. Conclusions

This study found a significant positive association between magnesium and systolic blood pressure, but not diastolic blood pressure, among patients with hypertensive crises. This positive association of serum magnesium with systolic blood pressure was maintained after adjusting for covariates. This study’s findings suggest a potential role of magnesium in blood pressure among patients with hypertensive crises. However, when considering the limitations and strengths of our study, we do not recommend changing current clinical practice regarding the monitoring of magnesium in patients with hypertensive crises. We recommend monitoring magnesium levels based on clinical necessity. We recommend experimental studies with larger samples be conducted to evaluate the role of serum magnesium and/or serum magnesium-modifying therapies in controlling or regulating blood pressure in patients with hypertensive crises. Future studies should also evaluate the role of serum calcium-modifying therapies in blood pressure control in patients with hypertensive crises.

## Figures and Tables

**Table 1:** Baseline characteristics (*N* = 293).

Demographic Variables	
Age (Mean ± SD years; (range))	56.70 ± 12.86 (19–97)
Sex, *n* (%)	Male: 146 (49.8%)
	Female: 147 (50.2%)
Race, *n* (%)	White: 62 (21.2%)
	Black/African American: 213 (72.7%)
	Asian: 3 (1.0%)
	Other: 15 (5.1%)
Hypertensive Crises Diagnosis, *n* (%)	Hypertensive Urgency: 220 (75.1%)
	Hypertensive Emergency: 73 (24.9%)
History of Diabetes mellitus, *n* (%)	Diabetic: 102 (34.8%)
	Non-diabetic: 191 (65.2%)
Body Mass Index (BMI) (Mean ± SD kg/m^2^; (range)) (*N* = 290)	30.59 ± 9.33 (16.70–69.10)
Use of Home Proton Pump Inhibitors (*N* = 259), *n* (%)	53 (20.5%)
Use of Home Blood Pressure Medications (*N* = 264), *n* (%)	227 (85.9%)
Use of Hospital Blood Pressure Medications, *n* (%)	283 (96.6%)
Use of Home Magnesium (Oral) (*N* = 292), *n* (%)	31 (10.6%)
Use of Hospital Magnesium (Intravenous) (*N* = 292), *n* (%)	95 (32.5%)
Serum Magnesium at Crises (Mean ± SD mg/dL; (range))	1.93 ± 0.36 (0.80–3.90)
Serum Calcium at Crises (Mean ± SD mg/dL; (range))	8.92 ± 0.92 (0.80–13.10)
Corrected Calcium at Crises (Mean ± SD mg/dL; (range)) (*N* = 207)	9.33 ± 0.90 (1.12–13.34)
Serum Potassium at Crises (Mean ± SD mg/dL; (range))	3.92 ± 0.64 (1.40–6.30)
Systolic Blood Pressure (Mean ± SD mmHg; (range))	194.2 ± 21.31 (136–265)
Diastolic Blood Pressure (Mean ± SD mmHg; (range))	113.7 ± 21.38 (53–180)

**Table 2:** Association of serum magnesium and other electrolytes with SBP at crises or at crises (*N* = 293).

	**SBP at Crises**		
**Variables**	**r**	**R^2^**	***p*-Value**
Serum Magnesium	0.143	0.020	0.014
Serum Calcium	0.187	0.035	0.001
Corrected Calcium (*N* = 207)	0.049	0.002	0.482
Serum Potassium	−0.076	0.006	0.195
	**DBP at Crises**		
**Variables**	**r**	**R^2^**	***p*-Value**
Serum Magnesium	0.033	0.001	0.570
Serum Calcium	0.090	0.008	0.124
Corrected Calcium (*N* = 207)	−0.011	0.000	0.873
Serum Potassium	−0.113	0.013	0.053

SBP—systolic blood pressure; DBP—diastolic blood pressure.

**Table 3:** Association of serum magnesium and SBP at crises or DBP at crises using linear model analysis (adjusted for covariates) (*N* = 293).

	**SBP at Crises**	
**Variables**	**β ± SE**	***p*-Value**
Serum Magnesium (per 1 mg/dL increase)	11.25 ± 4.67	0.017
	**DBP at Crises**	
**Variables**	**β ± SE**	***p*-Value**
Serum Magnesium (per 1 mg/dL increase)	2.56 ± 4.93	0.603

Adjusted for covariates—Age, sex, race, history of diabetes, BMI, use of proton pump inhibitors at home, use of blood pressure medications at home or hospital, use of oral magnesium at home, use of intravenous magnesium at hospital, serum calcium at crises, corrected calcium at crises, and serum potassium at crises.

**Table 4:** Association of best independent variables with SBP at crises using linear model analysis (adjusted for covariates) (*N* = 293).

	SBP at Crises	
Independent Variables	β ± SE	*p*-Value
Serum Magnesium (per 1 mg/dL increase)	11.25 ± 4.67	0.017
Serum Calcium (per 1 mg/dL increase)	9.50 ± 3.37	0.006
Corrected Calcium (per 1 mg/dL increase) (*N* = 207)	−7.82 ± 3.54	0.029
Use of Home Proton Pump Inhibitors	9.13 ± 3.85	0.019

Covariates in the mixed model—age, sex, race, history of diabetes, BMI, use of proton pump inhibitors at home, use of blood pressure medications at home or hospital, use of oral magnesium at home, use of intravenous magnesium at hospital, serum magnesium at crises, serum calcium at crises, corrected calcium at crises, and serum potassium at crises. *p*-values < 0.05 defined as significant for best independent variables.

**Table 5:** Association of best independent variables with DBP at crises using linear model.

	DBP at Crises	
Independent Variables	β ± SE	*p*-Value
Serum Calcium (per 1 mg/dL increase)	16.21 ± 3.56	<0.001
Corrected Calcium (per 1 mg/dL increase) (*N* = 207)	−14.12 ± 3.74	<0.001
Age	−0.53 ± 0.12	<0.001

Covariates in the mixed model—age, sex, race, history of diabetes, BMI, use of proton pump inhibitors at home, use of blood pressure medications at home or hospital, use of oral magnesium at home, use of intravenous magnesium at hospital, serum magnesium at crises, serum calcium at crises, corrected calcium at crises, and serum potassium at crises. *p*-values < 0.05 defined as significant for best independent variables.

**Table 6:** Association of serum magnesium at crises with SBP and DBP at different time points (*N* = 293).

	Serum Magnesium at Crises		
Variables	r	R^2^	*p*-Value
SBP at Crises	0.143	0.020	0.014
SBP Maximum (24 h)	0.104	0.011	0.074
SBP Minimum (24 h)	−0.034	0.001	0.563
DBP at Crises	0.033	0.001	0.570
DBP Maximum (24 h)	0.041	0.002	0.480
DBP Minimum (24 h)	0.021	0.000	0.726

**Table 7:** Association of serum calcium at crises with SBP and DBP at different time points (*N* = 293).

	Serum Calcium at Crises		
Variables	r	R^2^	*p*-Value
SBP at Crises	0.187	0.035	0.001
SBP Maximum (24 h)	0.134	0.018	0.022
SBP Minimum (24 h)	0.237	0.056	<0.001
DBP at Crises	0.090	0.008	0.124
DBP Maximum (24 h)	0.121	0.015	0.038
DBP Minimum (24 h)	0.183	0.033	0.002

**Table 8:** Association of corrected calcium at crises with SBP and DBP at different time points (*N* = 207).

	Corrected Calcium at Crises		
Variables	r	R^2^	*p*-Value
SBP at Crises	0.049	0.002	0.482
SBP Maximum (24 h)	−0.028	0.001	0.690
SBP Minimum (24 h)	0.256	0.066	<0.001
DBP at Crises	−0.011	0.000	0.873
DBP Maximum (24 h)	−0.000	0.000	0.996
DBP Minimum (24 h)	0.177	0.031	0.011

**Table 9:** Association of serum potassium at crises with SBP and DBP at different time points (*N* = 293).

	Serum Potassium at Crises		
Variables	r	R^2^	*p*-Value
SBP at Crises	−0.076	0.006	0.195
SBP Maximum (24 h)	−0.074	0.005	0.209
SBP Minimum (24 h)	−0.130	0.017	0.026
DBP at Crises	−0.113	0.013	0.053
DBP Maximum (24 h)	−0.065	0.004	0.265
DBP Minimum (24 h)	−0.175	0.031	0.003

## Data Availability

The data presented in this study are available upon reasonable request from the corresponding author (IfeanyiChukwu O. Onor) and with permission of University Medical Center New Orleans (formerly ILH). The data are not publicly available due to restrictions applied to the availability of these data by University Medical Center New Orleans (formerly ILH), which were used under license for the current study.
